# Pre-operative systemic therapy in locally advanced breast cancer: a single institution experience

**DOI:** 10.3332/ecancer.2009.161

**Published:** 2009-10-12

**Authors:** EM Ibrahim, AM Al-Gahmi, JM Zekri, SS Awadalla, TR Elkhodary, EE Fawzy, YA Bahadur, ME Elsayed, A Zeeneldin, RH Al-Ahmadi, AH Linjawi

**Affiliations:** 1 Department of Oncology, King Faisal Specialist Hospital and Research Center, Jeddah 21499, Kingdom of Saudi Arabia; 2 Research Center, King Faisal Specialist Hospital and Research Center, Jeddah 21499, Kingdom of Saudi Arabia; 3 Department of Surgery, King Faisal Specialist Hospital and Research Center, Jeddah 21499, Kingdom of Saudi Arabia

## Abstract

**Background:**

Locally advanced breast cancer (LABC) is common in developing countries and it frequently affects younger women. Patients do very poorly when treated by locoregional therapy alone; therefore, pre-operative systemic therapy (PST) is commonly used.

**Materials and methods:**

Medical records of 64 Saudi patients with LABC treated with PST in a single institution were retrospectively reviewed.

**Results:**

At diagnosis, most patients were young (median age 41 years), and had poor clinicopathological characteristics. Following surgery, complete pathologic response (pCR) in the breast was achieved in 13 patients (20%). Of 62 patients with known nodal status, 22 (34%) had negative axillary nodes. Presence of oestrogen receptor (ER) negative tumour was the only dependent variable that predicted pCR in the breast (p = 0.03). At a median follow-up of 42 months, the median progression-free survival (PFS) was 48 months (95% CI, 20–76 months) and the projected five-year overall survival (OS) was 68%. The recently published scoring system (Jeruss *et al* (2008) *J Clin Oncol* **26** 2 246–52), was the only variable that independently influenced PFS, while ER negative tumours and presence of lymphovascular space invasion were the only factors that adversely affected OS.

**Conclusions:**

despite the use of standard multi-modality approach in the management of patients with LABC, prognosis remains guarded.

## Introduction

Pre-operative systemic therapy (PST), initially used only for locally advanced breast cancer (LABC), has become more common for patients with operable disease [1–3]. In patients with operable breast cancer, randomized trials have demonstrated that PST and post-operative chemotherapy (using the identical agents and treatment schedules) result in the same disease-free (DFS) and overall survival (OS) [4]. It has also been shown that long-term outcome significantly correlates with both clinical and pathologic tumour response rates [5,6].

Recently, researchers at MD Anderson Cancer Center have developed scoring systems based on combined clinical and pathologic variables to define outcome for breast cancer patients treated with PST [7]. The study population was composed of 932 patients that were predominantly post- or perimenopausal with a median age of 50 years. The series included patients with LABC as well as patients with small primary tumour. In brief, the scoring systems assigned risk scores to clinical stage, pathologic stage, oestrogen receptor (ER) negative tumour, and grade III disease. A combined prognostic score ranging from 0 to 6 was developed (the higher score the worse was the prognosis). The study concluded that the scoring systems facilitated separation of the study population into more refined subgroups by outcome than the current staging system.

In Saudi Arabia like other developing countries, LABC is not only common but it also affects women at a much younger age, and it carries a distinctively poor outcome [8–11].

The primary objective of the current study was to describe the clinicopathologic features and to determine the outcome of patients with LABC treated in a single institution with PST. We also intended to test the utility of MD Anderson Cancer Center scoring systems in our patient population.

## Patients and methods

This retrospective study was approved by the Institutional Review Board of the King Faisal Specialist Hospital and Research Center. Data of newly diagnosed consecutive patients with locally advanced non-inflammatory invasive breast cancer (T2 > 4 cm, T3 or T4) confirmed on tru-cut biopsy and who received PST, were retrospectively reviewed. The database was locked on March 2009. Those with bilateral breast cancer, or documented evidence of metastatic disease, were excluded.

Staging procedures included complete history and physical examination, laboratory studies, bilateral mammography and mammary ultrasound, computerized tomography of the chest and abdomen, and radionuclide bone scan. ER and progesterone receptors (PR) were measured using standard immunehistochemistry (IHC), and the positive score was defined as greater than or equal to 10% of tumour cells demonstrating nuclear staining. HER-2 was graded as per the Dako HercepTest (Dako, Carpinteria, CA). For Her-2 +2 by IHC, HER-2 gene amplification was assessed by fluorescent *in situ* hybridization (FISH), using the Vysis method (Abbott Molecular, Inc., Des Plaines, IL). HER-2 positive tumours were those scoring 3+ by IHC or with > two copies of the HER-2 gene by FISH assay.

Clinical size of primary breast cancers and axillary nodes, if the latter were palpable, was determined separately before the administration of each cycle of PST and also before surgery. At each assessment, the product of the two greatest perpendicular diameters of the tumours in the breast and axilla was measured. Assessment of response was determined by clinical examination combined with mammography and ultrasonography. Women with dense breasts were also evaluated by magnetic resonance imaging.

## Therapy

Patients received various PST regimens at the discretion of the treating physician. Following the PST regimen, response was assessed both clinically and by repeating relevant imaging. Patients with minimal or no response after two to three cycles were considered for alternative chemotherapy regimens. After the last cycle, patients were scheduled to undergo conservative surgery or modified radical mastectomy upon discretion of the surgeon and according to patient preference guided by the clinical response. Axillary lymph node dissection to levels I and II, aiming for excision of at least ten lymph nodes, was to be performed.

While most patients received post-surgery radiotherapy to the chest wall or the conserved breast and the axilla, post-operative adjuvant chemotherapy or hormonal therapy was given according to the discretion of the oncologist guided by the pathological response and the relevant clinicopathological characteristics.

## Data source

A computerized database was created to capture hardcopy and electronic patient data. The following information was retrieved: patients’ demographic and clinical data, laboratory and radiological studies, disease characteristics and PST details including clinical response and toxicity. The database also included surgery details, axillary lymph node dissection and pathologic response. Also, captured were post-surgery further chemotherapy, hormonal therapy, radiation therapy, recurrence and survival.

## Definitions

Staging was defined according to the criteria determined by the International Union Against Cancer (UICC) [12], with group clinical and pathological staging according to the American Joint Committee on Cancer [13]. We adopted the criteria for LABC reported by Haagensen and Stout [14]. Best clinical response was assessed by physical and radiological examination and defined as complete response (cCR), partial response (cPR), stable disease (cSD), and progressive disease (cPD) according to response evaluation criteria in solid tumours [15]. This classification was also used to record the response of an axillary tumour to the PST regimen in patients who had clinically positive nodes at diagnosis. The development of a clinically suspicious ipsilateral axillary tumour during chemotherapy was considered as evidence of cPD in patients whose axilla was clinically negative when the first cycle of the PST was administered.

## Histopathology

A median of 15 sections of the mastectomy or lumpectomy specimen was assessed; these included sections from each quadrant, from the nipple–areola complex (if appropriate), from areas of suspicious or prior tumour involvement and from the axillary contents (median of seven sections).

Pathologic response was assessed in surgical specimens of mammary tissue and lymph nodes using Sataloff *et al* [16] and Chevallier *et al* [17] criteria. Complete pathological response (pCR) in the breast, was defined as disappearance of invasive disease in the breast by pathologic examination, while residual invasive disease of < 1 cm and ≥ 1 cm was considered for descriptive purpose only as micro- and macro-residual disease, respectively. pCR in axilla was defined as absence of positive lymph nodes by haematoxylin and eosin staining.

## Statistical methods

A two-sided Wilcoxon-Pratt test was used to compare tumour sizes before and after chemotherapy. To identify variables that predict pCR in the primary tumour, regression analysis was performed [18]. Overall survival (OS) was estimated from the date of starting PST to the date of last follow-up or death from any cause. Progression-free survival (PFS) was calculated from the date of definitive surgery until last contact; recurrence ‘local, regional or distant’; occurrence of contralateral breast cancer; occurrence of second primary cancer other than in the contralateral breast or death. Survival was estimated by applying the method of Kaplan and Meier [19], while the statistical procedure of Brookmeyer-Crowley was used to estimate the 95% confidence interval (CI) of median survival [20]. The log-rank test was used to assess the significance of unadjusted differences in survival [21]. Exploring variables for their independent prognostic effect on OS or PFS was carried out using the multivariate stepwise regression model of Cox and Oakes to compute hazards ratio (HR) [22]. Being one of the objectives of our study, we specifically examined the prognostic significance of the scoring system as reported by Jeruss *et al* and alluded to in the introduction [7]. In the survival analysis, we grouped patients according to their assigned risk score. Variables with p value ≤ 0.1 in the univariate analysis were tested for the multivariate model. In this process, the predictor with the highest level of statistical significance was used to introduce the model; other variables were then evaluated for further predictive information and added in turn, beginning with the variables with the highest level of statistical significance (i.e. the lowest p values) and continuing until the p value for the variable added exceeded 0.05. Continuous prognostic variables were also considered for inclusion in the model as dichotomous variables using various cut-off points only if they attained a p value of ≤ 0.1 in the univariate analysis. We also compared the survival functions for variables after stratifying for baseline differences in additional variables [23]. All tests of significance were two sided, and differences were considered statistically significant when P <0.05. We performed all data analyses using the SPSS Statistical Software Package (SPSS software v. 17.0; SPSS Inc., Chicago, IL, USA).

## Results

This retrospective analysis included the records of 64 patients, and they were all evaluable for efficacy and toxicity analysis. The median age was 41 years (range, 25–75). Patient and disease characteristics are depicted in Table 1. The median largest tumour diameter at diagnosis was 7.5 cm (range, 2–15 cm). Four (6%) and 60 (94%) patients had invasive lobular and invasive ductal cancer, respectively.

Table 2 shows the PST regimens and the adjuvant treatment given. Due to minimal or no response to initial anthracycline-based therapy, four patients required substitution of PST by taxane-platinum-based regimens. The median time from diagnosis to the initiation of PST was 24 days (95% CI, 32–69 days), and the median number of PST cycles was four (range, 2–8). Patients with HER-2 over-expression (seven patients) received neoadjuvant trastuzumab concomitantly with chemotherapy according to the MD Anderson protocol [7].

All patients were evaluable for the best clinical response (BCR). According to the pre-defined criteria, 18 (28%), 31 (48%), nine (14%) and six (10%) of the patients achieved cCR, cPR, cSD and cPD, respectively. The median time to the best clinical response was 2.7 months (95% CI, 2.6–4.6 months).

Following PST, patients underwent surgery (Table 2). The median time from starting therapy to surgery was 4.2 months (95% CI, 4–5.8 months). Despite a combined cCR and cPR rate of 76%, only 26 patients (41%) had conservative surgery. The pathologic response in the primary tumour was considered complete (pCR) in 13 (20%) of patients. On the other hand, residual invasive disease was microscopic (< 1 cm in diameter) and macroscopic (≥ 1 cm in diameter), in 18 (28%) and 33 (52%) patients, respectively. The median largest residual tumour diameter was 1.8 cm (range, 0–8). Compared with baseline assessment that downstaging was statistically significant (Table 3 depicts the pathologic data for the primary tumour and axillary lymph nodes. Of all 64 patients, eight (13%) achieved pCR in the primary tumour and in the axilla. None of the eight patients had residual non-invasive disease. The main clinicopathologic features of those eight patients were as follows: six patients (75%) were ≤ 50 years; four patients (50%) each had clinical stage IIB and IIIA, respectively; tumour grade II/III in 2/6 patients; lymphovascular space invasion −ve/unknown in 6/2 patients; ER+/ER− in 3/5 patients; PR+/PR− in 1/7 patients; and HER-2+/HER-2− in 4/4 patients. ER negative tumour was the only variable that independently predicted pCR in the breast (p = 0.03).

Seven patients who over-expressed HER-2 received neoadjuvant trastuzumab. Three patients achieved pCR in the primary tumour and the axilla. Two patients attained pCR in the primary tumour but with one positive lymph node in each patient. Of the remaining two patients, one had micro-residual disease and one had macro-residual disease. Neither of the latter two patients had positive axillary disease.

## Survival analysis

At a median follow-up of 42 months (95% CI, 34–49 months), 13 patients (20%) were dead, 15 (24%) were alive with evidence of disease and the remaining 35 (56%) were still alive with no apparent evidence of disease. All mortality events were attributed to progressive breast cancer or its related complications.

Disease recurrence was documented in 29 patients (45%). The first documented recurrence was local and/or regional, contralateral breast, contralateral lymph nodes, distant, distant and locoregional, in five, three, two, 18 and one patients, respectively. Of the eight patients, who attained pCR in the breast and axilla, three patients (38%) experienced relapse as compared with 26 of the 56 patients (46%) with less than pCR. The same three patients were the only patients who experienced relapse among all 13 patients with pCR in the breast. Of the seven patients, who received neoadjuvant trastuzumab, only one developed regional and distant relapse after 27 months. The median PFS for the series was 48 months (95% CI, 20–76 months) and the five-year PFS rate (±SE) was 48% (±7%).

**Figure 1:**
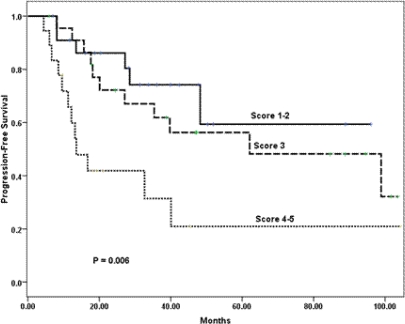
Kaplan-Meier estimates of progression-free survival of patients stratified into three risk groups according to the combined scoring system.

In a univariate analysis, the following variables prognosticated poor PFS and were subsequently tested in a multivariate model: age ≤ 35 years (p = 0.08); ER negative tumour (p = 0.07); tumour grade III (p = 0.001); pathologically positive lymph node (p = 0.008); extra-nodal extension (p = 0.07); and higher scoring system (p = 0.006) according to that proposed by Jeruss *et al* [7]. For the latter variable, we defined three distinctive risk groups (low-risk = 1–2 score, intermediate-risk = 3 score, and high-risk = 4–5 score). The Cox proportional hazards model identified higher score in the scoring system as the only variable that independently influenced PFS (p = 0.006) (Table 4 and Figure 1).

**Figure 2:**
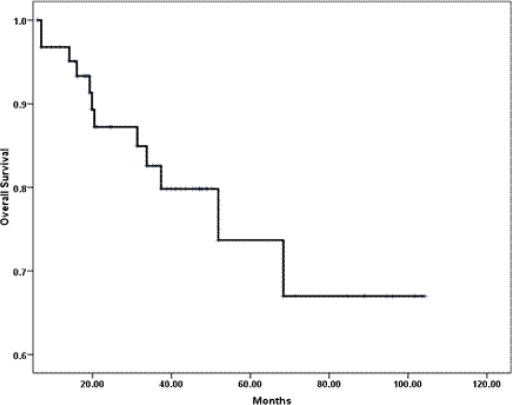
Kaplan-Meier estimate of overall survival.

The median OS has not been reached (Figure 2); however, the estimated five-year OS (±SE) was 68% (±9%). Disease-related mortality occurred in two of the eight patients who attained pCR in the breast and axilla. However, none of those who received neoadjuvant trastuzumab have died. In a univariate analysis, the following variables predicted poor OS and were subsequently tested in a multivariate model: tumour grade III (p = 0.02); ER− (p = 0.014); PR− (p = 0.03); lymphovascular invasion (p = 0.07); less than pCR (p = 0.06) and extra-nodal extension (p = 0.01). The scoring system showed no prognostic significance, and only ER negative tumours and the presence of lymphovascular space invasion were associated with an independent adverse effect on OS (Table 4).

PST was well tolerated with the expected treatment-related toxicity. There was no incidence of therapy-related mortality and no patient developed secondary malignancy other than contralateral breast cancer in three patients.

## Discussion

Patients with LABC do very poorly when treated by locoregional therapy alone. Such therapy favourably affects locoregional control, but most relapses are due to the development of distant metastases [24,25]. However, while PST regimens have been shown to have a favourable effect on the outcome of patients with LABC, the survival advantage of primary chemotherapy is yet to be shown [26–28].

In this series, there was significant prevalence of poor clinicopathologic features among patients. Nevertheless, PST achieved a significant downstaging of the primary tumour size and the clinical nodal status resulting in a high-clinical response rate of 76%, of which 28% was complete. However, only 20% and 28% of patients demonstrated pCR and microscopic residual invasive tumour, respectively. Moreover, of the 62 patients with known nodal status, 22 (35%) had negative axillary lymph nodes and, of those, eight patients (13%) had pCR in the breast and the axilla.

The regression model showed that ER negativity was the only variable that predicted pCR in the primary tumour. That finding is consistent with other published series that have shown that ER negative tumours tend to have a higher pathologic response rate to chemotherapy than ER positive disease [29–32].

Despite only seven patients with HER-2+ tumours receiving trastuzumab, three patients (45%) attained pCR in the primary tumour and the axilla. The achieved results are consistent with published studies. Incorporating trastuzumab with chemotherapy in the neoadjuvant setting in HER-2 over-expressing disease has uniformly demonstrated impressive pCR rate ranging from 39% to 67% [33–35].

At a median follow-up of 42 months (95% CI, 34–49 months), disease recurrence was documented in 45% of patients. The five-year PFS was 48%, and the median PFS was 48 months. Three of the seven patients who attained pCR in breast and axilla had relapsed. Reported series have shown a five-year recurrence rate in patients with a pCR ranging from 13% to 35% [3,36,37]. The five-year OS was projected as 68%, which is somewhat inferior to that reported in other series [38,39]; however, the attained OS was expected considering the poor clinicopathologic features of patients.

Interestingly, while the combined scoring system was developed on older patients with less advanced locoregional disease [7]; in our patients, the system was the only variable that distinctly classified patients into three risk groups with significantly different PFS rates. The scoring system, however, did not prognosticate OS. The lack of a prognostic effect of the scoring system on OS may be related to the few number of events. On the other hand, the Cox model identified ER tumour and the presence of lymphovascular space invasion as independent adverse prognostic variables for OS. In a large, recently published series, lymphovascular space invasion was a strong independent variable that predicted higher locoregional failure among patients with LABC [40].

The current series has several limitations. First, the series was relatively small; however, the series included a group of patients that largely had poor clinicopathologic characteristics. Second, various PST regimens have been used; however, all patients were offered the most active PST agents and received a uniform management strategy at a single institution.

In conclusion, this study described the clinical and pathological features, management strategy and outcome of young patients with LABC in a developing country. The study also identified predictor and prognostic variables that could influence outcome in a similar patient population. The prognosis of LABC remains guarded. The aggressive nature of LABC and the ability to measure drug effect *in vivo* indicate that more clinical trials of combining existing and newer biological agents with chemotherapy are needed [41,42].

## Figures and Tables

**Table 1:**
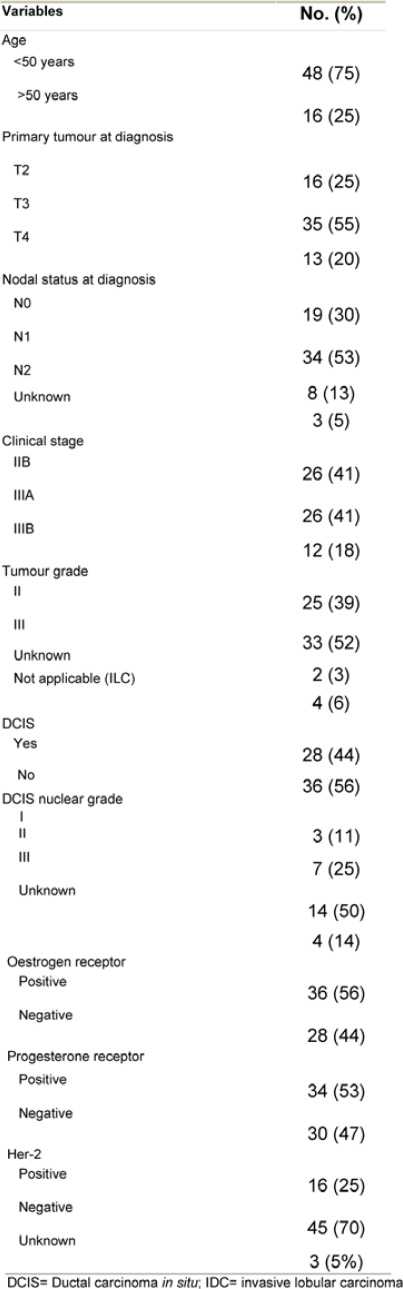
Patient and disease characteristics (64 patients)

**Table 2:**
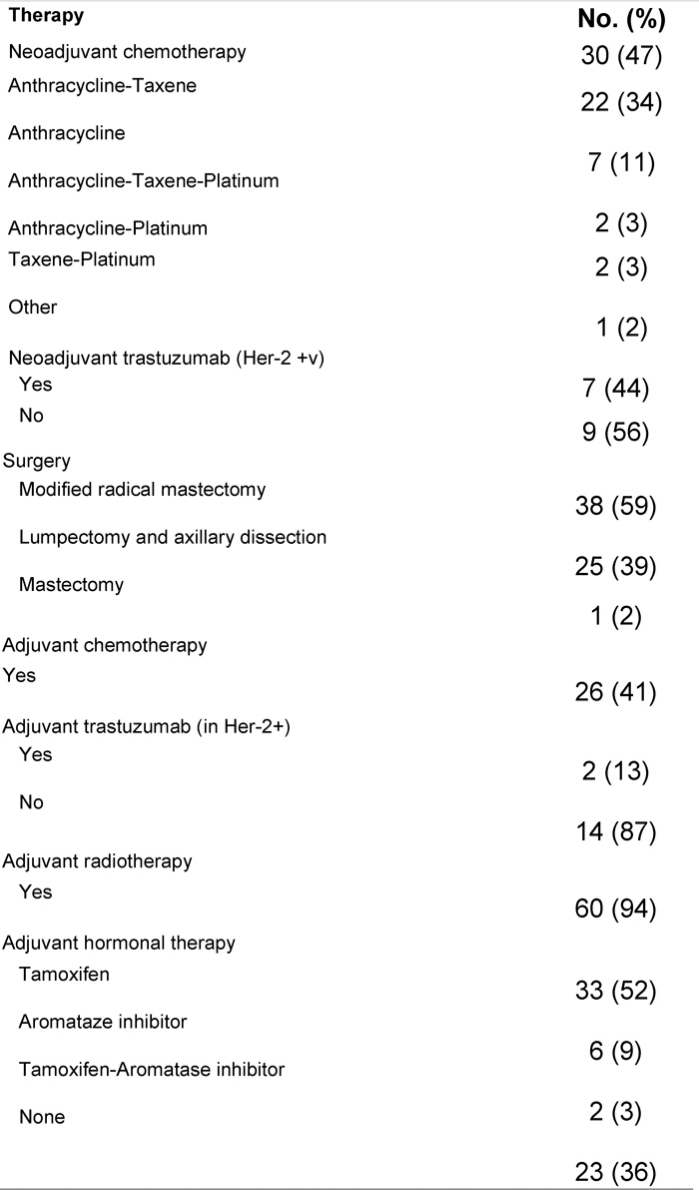
Neoadjuvant and adjuvant therapy

**Table 3:**
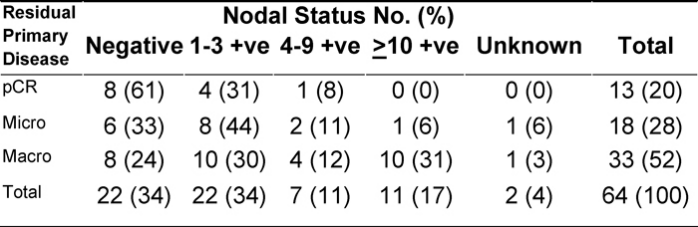
Relationship between pathologic responses of the primary tumour versus pathologic nodal status

**Table 4:**
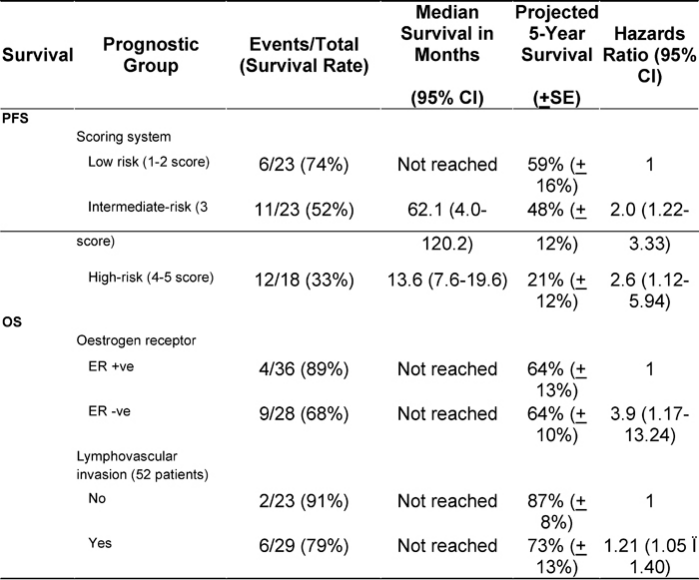
Multivariate analysis of prognostic variables for progression-free (PFS) and overall survival (OS)
